# Exanthematic Typhus

**Published:** 1858-12

**Authors:** 


### ART. III.—*Exanthematic Typhus.*

Prof. Wunderlich, in the second number of the Vierteljahrschrift,* for 1858, has collected and reported the observations of forty-nine cases of exanthematic typhus occurring in the Leipsic Hospital.

---

* Vierteljahrschrift für die Praktische Heilkunde. xv Jahrgang, 1858. Prag. Analekten, s. 14.

Notwithstanding this disease has repeatedly occurred in modern times as an epidemic, and its literature is extensive, and in many respects valuable, he yet finds in the representations relating thereto, many important deficiencies which must be filled up by careful and comprehensive observations.

He considers, in a particular manner, its propagation by contagion, which in the most of his cases (37) was evident, after the disease had been introduced by five strangers; the influence of age and antecedents, on the prognosis; the results of post-mortem sections, and the varieties of *abdominal* typhus which constantly occurred along with the *exanthematic!*

The contagion of exanthematic typhus shows a varying intensity at different times, and an incubation period of considerable latitude: at least the period between the infection and the outbreak of the first symptoms may occupy a period of more than two weeks; its tenacity is very considerable, and can operate at the end of half a year.

The disease may attack individuals of every race, of all ages, and of the most varying constitutions and conditions of health. A previous—and indeed, just preceding illness from *abdominal typhus*, diminishes the disposition not in the least,—whilst on the other hand, immediately after the *exanthematic*, the *enteric* can develop itself. Young and heretofore sound individuals, are incomparably less endangered by the approaching disease than older and hitherto feeble. Immoderate exertion before and at the commencement of sickening, increases the danger of the disease considerably. Three female attendants who sickened while attending devotedly to the sick in the hospital, all died. The seven convalescent and chronic patients, on the contrary, who likewise became affected while in the house, as also the only *clinicien* affected, passed through the disease favorably.

The commencement of the disease is for the most part definitely marked, and is indicated by prostration, headache, vertigo, heat in the head, loss of appetite, thirst, sometimes pain in the limbs, chills—though rarely by intense chills.

Its process is essentially typic, involving but few structures, and limited within few forms.

Its characteristics in the first week are,—its constant and rapid increase during the first half, and the slow decrease in favorable cases in the second half, or the transient remission on the seventh day; in seven cases, the continuance of high degree of temperature and cessation of the remission on the seventh day.

The peculiarities of the *second* week are,—the exacerbation in the commencement of the same, which is the rule in both mild and severe cases; the speedy return (in the first half) in mild cases; the non-appearance of the important morning remission even in most favorable cases; the remission between the twelfth and fourteenth days in mild, also in difficult—frequently even in fatal cases.

In the termination, the characteristics of convalescent cases consist in the rapid, or yet hastened defervescence, mostly between the thirteenth and seventeenth days, and through the entire subsidence of fever, at least from the second half of the third week; in *fatal* cases, by terminating either in the second half of the first week, or in the third week, and mostly after preceding lowering of temperature, a considerable increase of the same takes place in the agony of dissolution. In all cases, considerable elevation of temperature (3° or 4° over the normal) was attained, and this in such cases even, when the complex symptoms were unimportant An excessive increase of temperature gives an unfavorable prognosis, yet that before suffered in the exanthematic typhus, as also in most other affections, was especially more than in abdominal typhus. The morning and evening temperature shows only a slight difference; and this is of but little consequence in the first week, and first half of the second; somewhat more important in individual cases from the middle of the second until the close of the disease. A considerable sinking of temperature, during the continuation and increase of other grave phenomena, was a sure indication of collapse—the precursor of death.

The pulse-frequency (to which, as also to the temperature, is generally devoted a careful, very minute attention in W.'s clinics) is in general increased, and the degree of the same affords a stand point for the prognosis. When that reaches no more than ninety-six in the minute, the prognosis is very favorable, the abatement of the disease follows early. For the most part the pulse reaches a frequency of 110, and increases in convalescents even to 120 and over. A pulse, on the contrary, of over 132, in adults, with probability indicates death; also a rapid pulse, rapidly abating at a late period of the disease, indicates the same end. The greatest frequency of pulse is mostly on the tenth day. A considerable increase of pulse, with reduction of temperature is fatal. The different pulse-frequency between sitting and lying, is mostly unimportant; the double beating is almost wanting, or is slight and overlooked—as in abdominal typhus.

The roseolæ are present in all cases, but they show the greatest difference in relation to number and extent, and this is not in proportion to the gravity of the disease. The exanthems appear mostly from the third to the fifth, sometimes not till the seventh day. Sometimes relapses occur, but they are commonly slight. The extension of the roseolæ is by far more important than in enteric typhus; it appears commonly upon the extremities, very rarely the face. They continue a week and over, fade even before, or during the defervescence.

The cerebral symptoms are such as are observed in enteric typhus; in proportion to the grade of the disease they are, however, more violent, outlast not unfrequently the fever, and lose themselves first, after the normal temperature has been restored.

The tongue is found as in enteric typhus; the intestines are commonly affected, and exhibit phenomena very like those observed in enteric typhus; yet are they in general less lasting, not so perfectly formed, and less intensive. From the enteric phenomena alone, the differential diagnosis is not possible. The spleen is found as in abdominal typhus; its diminution (wiederveekleinerung) takes place with the defervescence, yet in convalescence even, it does not return to its original volume.

The diagnosis in the first days is wholly impracticable; it may with probability be conjectured, however, during the prevalence of exanthematic typhus, from the rapid accession of high febrile and brain symptoms. From the enteric typhus it distinguishes itself in the first week by the rapid increase of the symptoms, from the wanting double beat of the pulse, as also the pulse difference between lying and sitting; the flabbiness of the abdomen, and sometimes by brown stools. The diagnosis acquires a certainty in the middle and at the end of the first week, when the eruption abundantly appears, and the sometimes *papulous* form which distinguishes the exanthematic from the genuine typhus. The diagnosis is further confirmed if, in the second week, the high temperature makes no remission, or at least none of importance; and furthermore by this, that the enteric phenomena remain yet of trifling character. The diagnosis is finally established " with perfect certainty " through the mode of termination, since, in cases convalescing from exanthematic typhus, the extreme vacillation of temperature which characterizes the declining stage of enteric typhus, is wholly wanting; in fatal cases this termination is conditioned only through consuming fever, and not through any other local changes.(?)

In the prognosis, of special importance, are the antecedents of the patient; his age, his mode of living and relative soundness; the degree of previous muscular exertion, the timely care of the patient in the beginning of the disease; the absolute maximum height of temperature (33.3° R.'s and over, unfavorable); the continued increase of temperature in the second half of the first week, the remission on the seventh day (favorable); complications of every kind; lingering defervescence till after the commencement of the third week.

The section results of fourteen fatal cases are given. Dark cherry-red blood, moderate sized death spots, strong death stiffness, dry,—and less than in abdominal typhus,—dusky red-coloring of the muscles; integrity of the solitary glands and Peyer's conglomerate glands as also of the mesenteric glands; enlargement and softening of the spleen (contrary to the enteric typhus,) dark-green thick and viscid gall. The changes in the respiratory organs much as in enteric typhus.

